# A Cross-Sectional Study on Post-COVID-19 Menstrual Abnormalities in Women of Reproductive Age Group at a Tertiary Care Hospital

**DOI:** 10.1155/ogi/1771858

**Published:** 2025-03-25

**Authors:** Samyama Sagare Venkatesh, Malathi T., Manasa A. S. Gowda

**Affiliations:** ^1^Department of Obstetrics and Gynecology, Kempegowda Institute of Medical Sciences (KIMS), Bengaluru, Karnataka 560070, India; ^2^Department of General Medicine, Kempegowda Institute of Medical Sciences (KIMS), Bengaluru, Karnataka 560070, India

## Abstract

**Purpose:** This study aimed to evaluate the effect of COVID-19 infection and vaccination on all the menstrual cycle parameters in the women of reproductive age group, 18–45 years, at a tertiary care hospital.

**Methods:** A single-center, descriptive cross-sectional study was done from January 2, 2023, to June 24, 2023. Sampling was nonprobabilistic and purposeful. Participants were recruited via calls, in-person interviews, and online surveys. A total of 931 participants were recruited, of which 141 participants were eligible for the study. Descriptive statistics were performed for all variables. Pearson's chi-square test was done to compare categorical variables among different groups, and the Wilcoxon matched pair signed-rank test was done to compare the menstrual cycle patterns before and after COVID-19 infection and vaccination. Simple linear regression and multiple linear regression analysis were done wherever necessary. *p* < 0.05 was considered statistically significant.

**Results:** A total of 931 participants were recruited, of which 141 participants were eligible for the study. The median age was 29 years. Those who reported menstrual abnormalities were mainly of the age group 18–27 (*n* = 62, 44.0%), resided in an urban locality (*n* = 123, 87.2%), and were employed (full-time/part-time) (*n* = 57, 40.4%). Of the 42 participants with menstrual changes, 27 (64.3%) participants experienced changes post-COVID-19 infection before their first vaccination dose and 15 (35.7%) after the first vaccination dose. In this group, 15 (35.7%) continue to experience abnormalities in their cycles. Analysis showed that participants having severe COVID-19 symptoms were more likely to have an earlier onset of menstrual abnormalities (beta = −2.072, *p*=0.040). Participants with an above-normal BMI were more likely to have increased pain/cramps during menses (beta = 0.236, *p*=0.0.013). Participants who were students/employed (beta = −0.365, *p*=0.001) with an above-normal BMI (beta = 0.182, *p*=0.024) were more likely to experience increased mood swings/tension/irritability. On comparing the onset and duration of menstrual abnormalities in the post-COVID-19 infection and postvaccination groups, it was found that the latter group had a late-onset and short-term effect, while the former group had an early-onset and long-term effect on menses.

**Conclusion:** Our study shows that there is evidence of the onset of menstrual irregularities following COVID-19 infection and vaccination. The study revealed COVID-19 infection and vaccination influence menstrual cycles, the former posing a higher risk, but their effects on menstruation independent of one another are to be studied further.

## 1. Introduction

Coronavirus disease (COVID-19) is an infectious disease caused by severe acute respiratory syndrome coronavirus (SARS-CoV-2) [1]. The outbreak of the novel COVID-19 occurred in Wuhan, China, in December 2019, when it rapidly spread throughout the world, becoming a major disaster affecting public health [2]. As of October 4, 2023, the number of cases worldwide was estimated to be 771 million, among which India ranked third in the total number of cases, at 44 million [3]. The high incidence of cases highlights the need for further study of the disease.

Various studies show the novel virus can have a multiorgan impact. The clinical presentation of the disease may range from mild respiratory symptoms to severe progressive pneumonia, loss of appetite, loss of smell and taste, multiorgan failure, and even death [4, 5]. It is known to cause fatal respiratory diseases such as acute respiratory distress syndrome (ARDS) [2]. Hypercoagulability, cardiac injury, myocarditis, heart failure, and acute kidney injury (AKI) are a few of its cardiovascular and renal effects [2, 6]. There have been reports of orchitis in males, menstrual changes in females, and hormonal alterations in affected patients too [4, 7, 8]. One possible mechanism that might explain the pathogenesis of SARS-CoV-2 is its entry into cells through its receptor, angiotensin-converting enzyme-2 (ACE-2) [9]. Multiple organs show the expression of ACE-2 and, therefore, are a potential target for the virus [10]. ACE-2 is highly expressed in the ovary, fallopian tube, endometrium, and placenta of the female genital system, which means SARS-CoV-2 may even pose a risk to female reproductive health [11, 12].

Women who menstruate constitute approximately 49% of the total number of confirmed cases of COVID-19 infection [13]. The features of the menstrual cycle can act as indicators of a person's health and well-being [14]. It has been proven that women with irregular menstrual patterns are at a high risk of developing ovarian cancer, endometrial cancer, anemia, insulin resistance, cardiovascular disease, infertility, and premature menopause [15–19]. This can have a negative impact on the quality of life of women and cause significant morbidity. It also causes a socioeconomic burden on their families, health services, and society altogether [20–23].

A recent study was done to evaluate the existing scientific literature on changes in menstrual cycles post the COVID-19 pandemic. Thirty-three articles that found a positive association between the two were selected, which included worldwide data from China, India, Ireland, Turkey, Jordan, and Germany [23]. These studies evaluated the patients' sociodemographic data, sex hormone levels, ovarian reserves and reported changes in cycle duration, flow, and frequency. The findings suggested that menstrual abnormalities were often seen in patients with multisystem dysfunction of COVID-19, but these symptoms were often overlooked due to massive underreporting [23]. In addition to the data on postinfection menstrual abnormalities, studies in India have shown COVID-19 vaccinations like COVISHIELD and COVAXIN can cause changes in menstrual cycles in the form of longer and painful menses [24, 25]. Despite being ranked third in the total number of COVID-19 cases [3], there are not many studies based in India to evaluate SARS-CoV-2 infection and vaccination and their impact on the menstruation and ovarian function of women of childbearing age. Few studies suggest a link between COVID-19 infection and menstrual alterations and fewer link COVID-19 vaccinations. However, some show that neither affects menstrual cycles. Therefore, there is ambiguity and a lack of statistical knowledge surrounding the topic. The importance of female reproductive health has been growing, and attention to the effects of COVID-19 on the reproductive system has been called for globally [2, 4].

This study aims to evaluate the menstrual cycle parameters in women of reproductive age group and their causal relationship with COVID-19 infection and vaccination independent of one another. The collection and analysis of the sociodemographic, clinical, and laboratory characteristics of each participant was done. We evaluated the effects of the infection, vaccination, and medical histories on their menstrual cycles and assessed the onset and duration of menstrual abnormalities.

This study can help expand on the existing research on the postacute sequelae of SARS-CoV-2 infection and vaccination and its effects on women's reproductive health. It can provide further evidence on the incidence, onset, risk factors, severity, and duration of menstrual changes and provide scope for improved treatment options. Educating and increasing awareness among women about these findings can help them better understand their reproductive health, know when to seek medical care, and lessen any anxiety caused by these menstrual abnormalities.

## 2. Materials and Methods

### 2.1. Participants

A single-center, descriptive cross-sectional study was done on participants of reproductive age group with a history of confirmed COVID-19 at Kempegowda Institute of Medical Sciences (KIMS), Bangalore, from January 2, 2023, to June 24, 2023.

### 2.2. Inclusion Criteria

Inclusion criteria were as follows: (1) age category 18–45 years; (2) participants who identify as a woman and menstruate; (3) history of confirmed COVID-19 infection diagnosis prior to their vaccination first dose; and (4) interval between COVID-19 infection and vaccination first dose should be a minimum of 6 months.

### 2.3. Exclusion Criteria

Exclusion criteria were as follows: (1) pregnant or lactating at the time of infection or vaccination; (2) history of pregnancy within 3 months of COVID-19 infection; (3) history of pre-existing menstrual abnormalities or comorbidities directly influencing their menstrual cycles at the time of infection (thyroid disorders/gynecological disorders/OCP or IUD use/iron profile/Vit B12/anticoagulants); and (4) history of hysterectomy post-COVID-19 infection.

### 2.4. Data Collection

Sampling was nonprobabilistic and purposive. Participants were recruited via calls, in-person interviews at the outpatient clinics, and online surveys circulated on social media (WhatsApp and Instagram). We recruited 931 participants, of which 141 participants were eligible for the study.

The questionnaire consisted of 43 questions split into six sections: (1) participant identity and sociodemographic questions; (2) COVID-19 infection and vaccination history; (3) menstrual cycle patterns before COVID-19 infection; (4) menstrual cycle patterns after COVID-19 infection/vaccination first dose; (5) medical history before and after the diagnosis of COVID-19; and (6) laboratory investigations performed after the diagnosis of COVID-19.

Temporal relationship was established taking their COVID-19 diagnosis as the primary event. The selected participants had a history of COVID-19 infection prior to their vaccination first dose. Only participants having a minimum interval of 6 months between their COVID-19 infection diagnosis and vaccination first dose were selected, as previous studies showed a median interval of 3–6 months between COVID-19 infection and onset of menstrual irregularities [26, 27]. The higher end of the interval was taken as the cutoff, thus giving adequate time for participants to observe any changes in their menstrual cycles and speculate the cause [24].

Medical history was assessed for other independent causes of menstrual abnormalities such as a history of pregnancy/lactation/thyroid disorders/gynecological disorders (fibroid, PCOS, endometriosis, pelvic inflammatory disease)/OCP or IUD use/anticoagulant use/iron profile/Vit B12 levels and was factored out. The cause of menstrual changes was attributed to COVID-19 infection in those who experienced changes postinfection before vaccination first dose. The cause of menstrual changes was attributed to COVID-19 vaccination in those who experienced changes postvaccination first dose.

### 2.5. Definitions

All the participants were confirmed to have tested positive for COVID-19 according to the guidelines provided by the Ministry of Health and Family Welfare (MoHFW), India. A case should be confirmed as COVID-19 with a positive reverse transcription polymerase chain reaction (RT-PCR) test or SARS-CoV-2 antigen–rapid antigen test (RDT) test meeting either the probable case definition or suspect criteria.

The participants' body mass index (BMI) was categorized based on the Asia–Pacific Classification of BMI.

Participants' severity of COVID-19 infection was classified based on the guidelines set by the MoHFW, India: (1) mild: mild clinical symptoms without shortness of breath or hypoxia (normal SpO2); (2) moderate: pneumonia, dyspnea, and/or hypoxia, SpO2 90%–93%, respiratory rate more or equal to 24/minute; (3) severe: severe pneumonia or signs of ARDS, SpO2 < 90%.

Menstrual cycles were assessed based on the standardized parameters set by the International Federation of Gynaecology and Obstetrics (FIGO) on Abnormal Uterine Bleeding (Classification 1). AUB is defined as bleeding from the uterus, which differs in frequency, regularity, duration, or volume from normal uterine bleeding, in the absence of pregnancy (FIGO). This included the following parameters:

1. Frequency: Absent (no bleeding); infrequent (> 38 days); normal (≥ 24 to ≤ 38 days); frequent (< 24 days)
2. Duration: Normal (≤ 8 days); prolonged (> 8 days)
3. Flow volume: Light, normal, heavy
4. Presence of clots/pain or cramps/mood swings/irritability/tension or anxiety/appetite changes/sleep disturbance.

Obstetric score was written as P (parity), L (live births), and A (abortions).

Parity denotes the number of previous pregnancies beyond the period of viability (24 weeks of gestation). L denotes the number of live births. Abortion denotes the number of pregnancies that were terminated before 20 weeks of gestation (due to the medical termination of pregnancy/miscarriages/ectopic pregnancy).

Normal hemoglobin levels were taken as 12–16 gm/dL in adult women. Normal thyroid-stimulating hormone (TSH) levels were taken as 18–29 yrs = 0.5–2.34 mIU/L and 30–49 yrs = 0.5–4.0 mIU/L.

Normal serum iron ferritin levels for adult females were taken as 24–307 ng/mL.

Normal vitamin B12 levels in adult females were taken as 160–950 pg/mL. Normal total blood cholesterol levels were taken as 125–200 mg/dL.

### 2.6. Statistical Analysis

Analysis was performed using IBM SPSS Statistics for Windows, Version 29.0 (Armonk, NY: IBM Corp).

Descriptive statistics were performed for all variables. Categorical variables were described as frequencies (percentages). Continuous variables were presented as mean ± SD. Pearson's chi-square test was used to compare the percentages of categorical variables among different groups. Wilcoxon matched pair signed-rank test was done to compare menstrual cycle patterns before and after COVID-19 infection. This test compares the means of two related samples (paired data) when the data are not normally distributed. Simple linear regression was done to screen the independent variables that may affect the menstrual cycle parameters followed by multiple linear regression analysis for those variables that were statistically significant. For all statistical tests, *p* < 0.05 was considered statistically significant.

### 2.7. Ethical Consideration

This study was approved by the Institutional Ethics Committee of KIMS, Bangalore (Ref No: KIMS/IEC/A020/M/2022).

## 3. Results

A total of 141 participants were included in the study. All the participants identified as women. The median age was 29 years, and their sociodemographic and clinical data are mentioned in Table 1.

Most participants belonged to the age group 18–27 years (*n* = 62; 44.0%). Thirty-nine (27.7%) belonged to the age group 28–37, and 40 (28.4%) belonged to the age group 38–45. One hundred and twenty-three participants (87.2%) resided in an urban locality, while 18 (12.8%) of them resided in nonurban areas. At the time of survey, 57 (40.4%) participants were employed, 46 (32.6%) were homemakers, and 38 (27.0%) were students. Fifty-seven (40.4%) participants had a BMI in the normal range (18.5–22.9). Forty (28.4%) were overweight, 34 (24.1%) were preobese, 6 (4.3%) were obese, and 4 (2.8%) were underweight. Majority of the participants had a mixed diet (*n* = 83, 58.9%).

The participants had COVID-19 symptoms for a mean duration of 8.62 days. Around half of them recovered within 7 days (*n* = 69, 48.9%), while 60 (42.6%) within 2 weeks, and the rest took more than 2 weeks for recovery. One hundred and six (75%) participants had mild COVID infection, 28 (19.9%) were moderately ill, and only 7 (5%) participants had severe COVID infection. Fifteen (10.6%) of the total participants were reinfected within 3 years of the first diagnosis.

97.2% (*n* = 135) of the participants were completely immunized. The majority were vaccinated with COVISHEILD (*n* = 108, 76.7%), 29 (20.6%) received COVAXIN, 2 (1.4%) received other vaccines, and 2 (1.4%) were not immunized.

Forty-two (29.8%) of the total 141 participants showed changes in their menstrual cycles. Twenty-seven (64.3%) participants experienced menstrual changes post-COVID infection before their vaccination first dose and 15 (35.7%) postvaccination first dose. In this group, 15 (35.7%) continue to experience abnormalities in their cycles.

**Figure 1:**
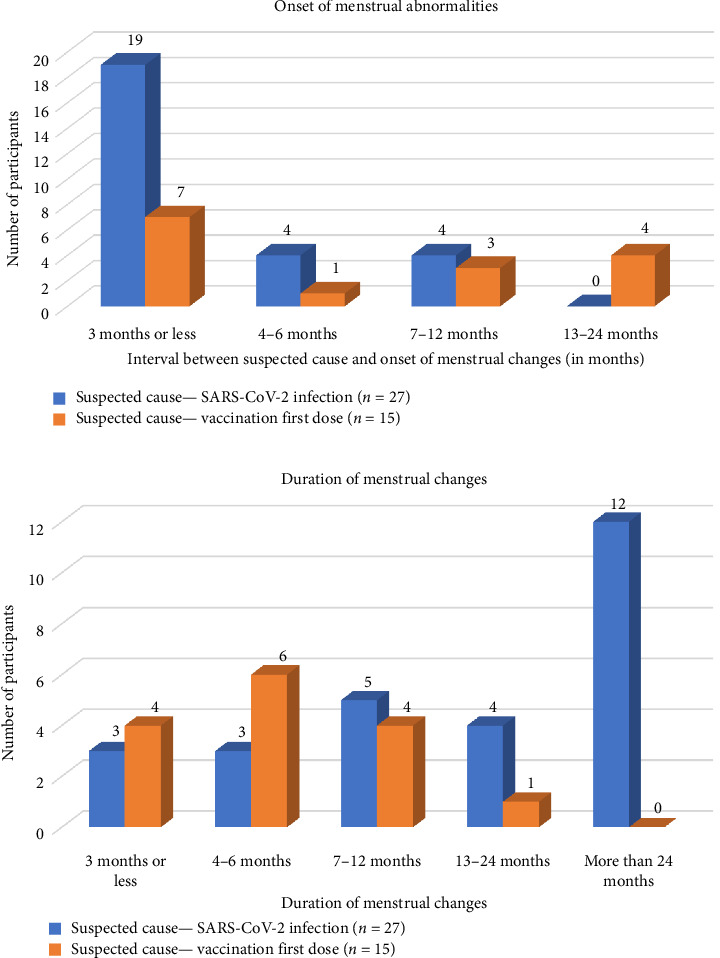
Interval and duration of menstrual abnormalities.

The median duration between the COVID-19 diagnosis and onset of menstrual changes in the former group (*n* = 27) was 2 months with menstrual abnormalities lasting an average of 19.48 months. The median duration between first vaccination dose, and the onset of menstrual changes in the latter group (*n* = 15) was 4 months with menstrual abnormalities lasting an average of 6.88 months (Tables 1 and 2 and Figure 1).

Of those participants found to have menstrual abnormalities, 64.3% (*n* = 27) of the participants have normal hemoglobin levels and 8 (19%) have reduced Hb count; 33 (78.6%) participants have normal TSH levels and 3 (7.1%) have increased TSH levels; 5 (11.9%) have a history of controlled hypothyroidism upon treatment, 2 (4.8%) have a history of uncontrolled hypothyroidism; 1 (2.4%) has a history of untreated hyperthyroidism; 1 (2.4%) has a history of treated PCOS; 1 (2.4%) had undergone blood transfusions due to severe anemia; 15 (35.7%) have no history of pregnancy; and 27 (64.3%) have a history of pregnancy, with a majority having had two pregnancies in the past with two living children (P2L2A0, *n* = 15, 35.7%), of which 26 occurred before 2020.

**Figure 2:**
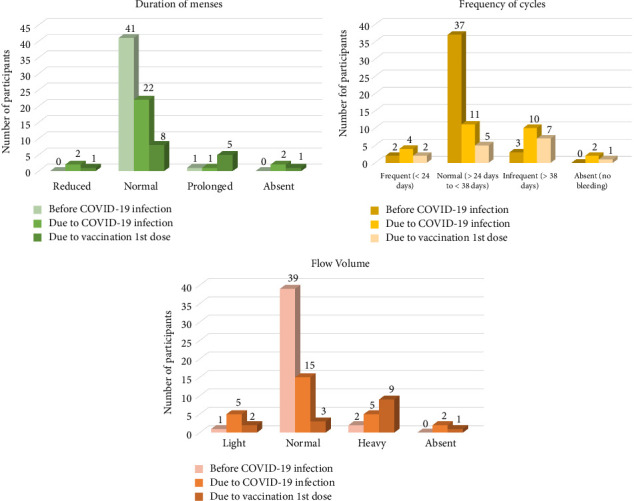
Changes in menstrual parameters.

The menstrual cycle patterns of all the women before COVID-19 infection and after COVID-19 infection/vaccination are listed in Table 3 and Figure 2 per the FIGO Abnormal Uterine Bleeding Classification 1. Among those 42 participants with menstrual changes, the duration of menstruation was prolonged for six (14.3%) participants, reduced for three (7.1%), and completely absent for three (7.1%). Seventeen (40.5%) had irregular cycles, and six (14.3%) had frequent cycles. Fourteen (33.3%) participants experienced heavier flow, while 7 (16.7%) experienced lighter flow than their normal. 6 (14.3%) had an increased number of clots, 24 (57.1%) experienced increased pain/cramps, 6 (14.3%) experienced an increase in mood swings/tension/irritability, and 8 (19.0%) had more appetite problems and sleep disturbances than normal.

On performing the chi-square test, a significant correlation was found between the menstrual cycle parameters and the onset of menstrual changes (*p*=<0.001) and the duration of menstrual abnormalities (*p*=<0.001). A significant correlation was also found between the participant's BMI and flow volume changes (*p*=0.007).

On performing the Wilcoxon signed-rank test between the parameters of women's menstrual cycles before and after COVID-19 infection, a significant difference in the medians was found in the parameters: duration (*p*=0.003), frequency (*p*=0.005), flow volume (*p*=0.028), pain/cramps (*p*=0.001), and premenstrual symptoms like appetite changes (*p*=0.008) and sleep disturbances (*p*=0.009).

The effect of independent variables such as age, occupation, BMI, and diet on various menstrual cycle parameters was investigated using simple linear and multiple linear regression analysis (Table 4). The results showed that participants having severe COVID-19 symptoms were more likely to have an earlier onset of menstrual abnormalities (beta = −0.186, *p*=0.040). Participants with an above-normal BMI were more likely to have increased pain/cramps during menses (beta = 0.236, *p*=0.013). In addition, those participants who were students/employed (beta = −0.365, *p*=0.001) with an above-normal BMI (beta = 0.182, *p*=0.024) were more likely to experience increased mood swings/tension/irritability. Women who were homemakers were less likely to experience sleep disturbances during menses (beta = −0.324, *p*=0.009).

## 4. Discussion

Since December 2019, the outbreak of the novel COVID-19 has become a multifaceted challenge to healthcare systems around the world. Under the influence of multiple factors such as advanced age, occupation, high BMI, diet, and history of medical illnesses, it has caused significant morbidity and mortality at the time of infection with critical sequelae [2].

Age and sex are two factors that are known to play an important role in COVID-19 progression [2, 4]. Old age is one of the highest risk factors for COVID-19 infection attributed to higher ACE-2 expression, immune dysregulation, and poor nutrition, which makes them more susceptible to viral complications and mortality. It is also observed that the incidence of COVID-19 in men is higher than in women. The decreased susceptibility of females to viral infections could be attributed to the protection from X chromosome and sex hormones, which play an important role in innate and adaptive immunity [2].

Since the beginning of the COVID-19 pandemic, there have been accumulating studies indicating that women have experienced menstrual changes, including altered menstrual duration, frequency, regularity, and volume, increased dysmenorrhea, and worsened premenstrual syndrome (PMS) [2, 23, 28].

Normal menstrual cycles are an indicator of the normally functioning hypothalamic–pituitary–gonadal (HPG) axis and are a vital sign of a woman's health [29]. Normal variation exists within women over their lifespan caused by an interaction of various factors such as parity, BMI, diet, occupation, and smoking [30]. In addition, menstrual cycle features such as volume, pain, distress, and PMS are subjective and self-perceived symptoms vary from one person to another [31, 32].

Taking those variations into consideration, our study had a total of 141 eligible participants, of which 42 (29.8%) had reported menstrual abnormalities. This fraction affected is comparable to studies done previously [26, 28, 33]. Those who reported menstrual abnormalities were mainly of the age group 18–27, resided in an urban locality, and were employed (full-time/part-time). These results are consistent with a study done in Spain, suggesting that menstrual alterations are associated with long COVID-19 in those younger than 25 years of age and employed (full-/part-time) [34]. There is a possibility that urban/educated participants reported more changes in their menstrual cycles due to their access to media/news about COVID-19 infection and vaccination altering menstrual patterns. This may have subjected them to a certain amount of bias while reporting their menstrual changes [20].

The mean duration of COVID-19 infection symptoms was 8.62 days, and the median age of participants was 29 years. In our study, the incidence of menstrual changes was higher in participants with mild COVID-19 symptoms and a recovery time of > 8 days. Participants who had severe COVID-19 symptoms were more likely to have an early onset of menstrual abnormalities, similar to the findings of a study done in India [24].

64.2% (*n* = 27) of the participants had an above-normal BMI (> 22.9), and it is well documented that menstrual abnormalities can be associated with females having a higher BMI [28, 30]. A high BMI indicating excess body fat can disrupt hormone balance, particularly estrogen and progesterone, leading to irregular ovulation and menstrual abnormalities such as irregular cycles, heavy bleeding, and pain. Upon analysis, it was found that participants with an above-normal BMI (overweight/preobese/obese) were more likely to have increased pain/cramps during menses, thereby supporting a similar study done in Arizona [28].

Type of occupation has been known to affect menstrual cycles post-COVID [34]. Those participants who were employed/students with an above-normal BMI were likely to experience increased mood swings/tension/irritability and women who were homemakers were less likely to experience sleep disturbances post-COVID. These findings are consistent with the study done in Spain [34].

Diet plays a major role in defending against microbial infections including COVID-19. A diet rich in Vitamins B1, B6, and B12, folate, Vitamin D, selenium, iron, and zinc is known to improve immune defenses, thereby reducing the severity of infections like COVID-19 [35, 36]. Iron and Vitamin B12 levels were assessed in this study to rule out any prior history of menstrual abnormalities and risk factors for COVID-19 infection. Their deficiencies are known to cause anemia and subsequently suppress immunological response [37]. Heavy menstrual bleeding is also a known contributor to iron deficiency anemia [38]. 57% (*n* = 24) of participants consumed a mixed diet, and majority had normal iron and Vitamin B12 levels, but no significant correlation (*p*=>0.5) was found between these factors and the severity of COVID and post-COVID menstrual abnormalities in our study.

Of the 42 participants with menstrual abnormalities, 27 reported menstrual changes postinfection (COVID-19 positive before the first vaccination dose) and 15 reported menstrual changes postvaccination first dose. The median duration between the COVID-19 diagnosis and onset of menstrual changes in the former group (*n* = 27) was 2 months with menstrual abnormalities lasting an average of 19.48 months. The median duration between first vaccination dose and the onset of menstrual changes in the latter group (*n* = 15) was 4 months with menstrual abnormalities lasting an average of 6.88 months. A similar study done in Turkey [26] stated that menstrual changes were observed within the first three cycles (median) following infection/vaccination. These results are comparable to similar studies done in India and the United States, which suggest that vaccines have a late-onset and short-term effect, while the infection has an early-onset and long-term effect on menses [24, 26, 27].

The mechanism behind the difference in the prevalence of menstrual abnormalities postinfection could be due to the persistent binding of virus to the ACE-2 receptors in the ovary, leading to reduced ovarian reserve and ovulation defects like irregular menses [26]. The faster onset and longer duration of symptoms post-COVID-19 infection can cause ongoing abdominal pain, pelvic discomfort, and irregular cycles. It can also increase anxiety about health, fertility, and possible long-term effects.

Of the participants with menstrual abnormalities, those who received COVISHIELD (*n* = 36, 85.7%) were more than those who received COVAXIN (*n* = 6, 14.3%). But no significant correlation was found between the type of vaccine and its effect on menstrual parameters (*p*=>0.05) [25].

Among those participants with menstrual abnormalities due to COVID-19 infection, the main changes seen were infrequent cycles (*n* = 10), heavy flow with clots (*n* = 5), and painful menstruation (*n* = 16). They also experienced more appetite changes (*n* = 6) and sleep disturbances (*n* = 6) [7, 26, 28, 33]. While participants with menstrual abnormalities due to vaccination experienced prolonged cycles (*n* = 5), infrequent cycles (*n* = 7), heavy flow (*n* = 9), and painful menstruation (*n* = 8) [24–27, 39]. When compared to individuals who are vaccinated, a history of COVID-19 disease is significantly associated with an increased risk of reporting longer menses duration, irregular menses, and heavier bleeding, which is in line with previously done studies [40, 41]. Twenty-seven of the 42 participants completely recovered, while 15 still experienced persistent changes [7]. This is similar to previous studies that suggest that the menstrual changes are most likely transient and reversible [24, 27, 42].

The mechanisms underlying the menstrual irregularities caused due to vaccines are not yet established. However, one hypothesis states that vaccines may cause strong immune reactions and stress that can temporarily affect the hypothalamic–pituitary–ovarian axis [29, 43]. Another hypothesis is that vaccine-mediated thrombocytopenia/low platelet count can cause heavy bleeding. Similar changes have also been reported following vaccinations such as measles–mumps–rubella, Hepatitis A and Hepatitis B, influenza, chickenpox, and diphtheria–tetanus–acellular pertussis [26].

## 5. Conclusion

In our study, there is evidence to suggest the onset of menstrual irregularities occurs following COVID-19 infection and vaccination. The main changes seen were infrequent cycles, heavy flow with clots, and painful menstruation. The study revealed that COVID-19 infection and vaccination influence menstrual cycles, the former posing a higher risk, but their effects on menstruation independent of one another are to be studied further.

## 6. Limitations

There are a few limitations in our study. First, due to a relatively small sample size, the eligible sample included is not representative of the entire Indian population. Second, the study design does not allow us to establish causality. Third, due to the study being retrospective in nature, the participants were susceptible to recall bias. Fourth, the study was of questionnaire type limited to a single center, obtained via social media, calls, or in-person interviews, which may have introduced selection bias and interviewer bias. Fifth, due to the time constraints of the study, a limited sample size was obtained. Sixth, participant's perceptions, expectations, or bias toward the impact of COVID-19 infection or vaccination on their menstrual cycles was not assessed in this study. Last, this study did not assess the stress and psychological status of the participants, which may have been a confounding factor as it is known to significantly influence menstrual cycles.

## 7. Recommendations

Women are less likely to seek medical attention for menstrual abnormalities that are transient and minor. Numerous studies have correlated the effect of COVID-19 infection and vaccination on menstrual cycles, but more research is yet to be done on their long-term effects on fertility and women's reproductive health. It is critical to raise awareness on menstrual alterations following COVID-19 infection and immunization and to warn and alert women about this concern. Studies like this one can help educate and increase awareness among women about their reproductive health and can also help lessen any anxiety caused by monthly abnormalities.

This study expands on previous cohort studies that mostly used mRNA vaccines. It adds to the existing knowledge of the effects of adenovirus-vectored (COVISHIELD) and whole inactivated virus-based (COVAXIN) COVID-19 vaccines. It provides scope for further research into the side effects of these vaccines administered in India.

## Figures and Tables

**Table 1:** Sociodemographic characteristics, self-perceived COVID-19 details, COVID-19 vaccination details, medical history details, and laboratory investigation details.

**Variable**		**SARS-CoV-2-positive participants without menstrual changes after infection (*n* = 99; 70.2%)**	**SARS-CoV-2-positive participants with menstrual changes after infection (*n* = 42; 29.8%)**	**Total**
Age	Mean (SD) Median (in years)			30.62 ± 8.4 29

Age	18–27	46 (46.5%)	16 (38.1%)	62 (44.0%)
	28–37	27 (27.3%)	12 (28.6%)	39 (27.7%)
	38–45	26 (26.2%)	14 (33.3%)	40 (28.4%)

Locality	Nonurban	13 (13.1%)	5 (11.9%)	18 (12.8%)
	Urban	86 (86.9%)	37 (88.1%)	123 (87.2%)

Occupation	Student	30 (30.3%)	8 (19.0%)	38 (27.0%)
	Employed	37 (37.4%)	20 (47.6%)	57 (40.4%)
	Homemaker	32 (32.3%)	14 (33.3%)	46 (32.6%)

BMI	18.5 or less	4 (4.0%)	0 (0.0%)	4 (2.8%)
	18.5–22.9	42 (42.4%)	15 (35.7%)	57 (40.4%)
	23.0–24.9	31 (31.3%)	9 (21.4%)	40 (28.4%)
	25.0–29.9	19 (19.2%)	15 (35.7%)	34 (24.1%)
	30.0 and above	3 (3.0%)	3 (7.1%)	6 (4.3%)

Diet	Vegetarian	40 (40.4%)	18 (42.9%)	58 (41.1%)
	Mixed	59 (59.6%)	24 (57.1%)	83 (58.9%)

Method of testing	RT-PCR	88 (88.9%)	42 (100%)	130 (92.2%)
	Rapid antigen test (RAT)	8 (8.1%)	0 (0.0%)	8 (5.7%)
	Other	3 (3.0%)	0 (0.0%)	3 (2.1%)

Duration of COVID-19 infection (in days)	Mean (SD)			8.62 ± 4.71

Duration of COVID-19 infection	7 days or less	51 (51.5%)	18 (42.%)	69 (48.9%)
	8–14 days	40 (40.4%)	20 (47.6%)	60 (42.6%)
	More than 14 days	8 (8.1%)	4 (9.5%)	12 (8.5%)

Severity of COVID-19 infection	Mild	79 (79.8%)	27 (64.3%)	106 (75.0%)
	Moderate	17 (17.2%)	11 (26.2%)	28 (19.9%)
	Severe	3 (3.0%)	4 (9.5%)	7 (5.0%)

Reinfection occurred	No	91 (91.9%)	35 (83.3%)	126 (89.4%)
	Yes	8 (8.1%)	7 (16.7%)	15 (10.6%)

COVID-19 vaccination doses	Not vaccinated	2 (2.%)	0 (0.0%)	2 (1.4%)
	One dose received	0 (0.0%)	2 (4.%)	2 (1.4%)
	Two doses received	76 (76.8%)	33 (78.6%)	109 (77.3%)
	Doses with booster received	21 (21.2%)	7 (16.7%)	28 (19.9%)

Vaccine received	Not vaccinated	2 (2.0%)	0 (0.0%)	2 (1.4%)
	COVISHIELD	72 (72.7%)	36 (85.7%)	108 (76.6%)
	COVAXIN	23 (23.2%)	6 (14.3%)	29 (20.6%)
	Other	2 (2.0%)	0 (0.0%)	2 (1.4%)

Interval between COVID-19 infection (suspected cause) and onset of menstrual changes (in months)	Mean (SD) Median			3.25 2

Interval between vaccination first dose (suspected cause) and onset of menstrual changes (in months)	Mean (SD) Median			7.86 4

Duration of menstrual changes postinfection (in months/cycles)	Mean (SD) Median			19.48 24

Duration of menstrual changes postvaccination first dose (in months/cycles)	Mean (SD) Median			6.88 5

Recovered or persistent menstrual changes	Persistent		15 (35.7%)	
	Recovered		27 (64.3%)	

Menstrual abnormalities started before/after first dose of vaccination	Before		27 (64.3%)	
	After		15 (35.7%)	

Hemoglobin (gm/dL)	Not done	20 (20.2%)	7 (16.7%)	27 (19.1%)
	Yes, normal	71 (71.7%)	27 (64.3%)	98 (69.5%)
	Yes, reduced	8 (8.1%)	8 (19.0%)	16 (11.3%)

Thyroid (TSH) levels	Not done	29 (29.3%)	6 (14.3%)	35 (24.8%)
	Yes, normal	70 (70.7%)	33 (78.6%)	103 (73.0%)
	Yes, increased	0 (0.0%)	3 (7.1%)	3 (2.1%)

Ultrasound (USG)	Not done	64 (64.6%)	21 (50.0%)	85 (60.3%)
	Yes, normal	32 (32.3%)	20 (47.6%)	52 (36.9%)
	Fatty liver	0 (0.0%)	1 (2.4%)	1 (0.7%)
	Mild polycystic ovary	1 (1.0%)	0 (0.0%)	1 (0.7%)
	Multiple fibroids and enlarged uterus	1 (1.0%)	0 (0.0%)	1 (0.7%)
	Spina bifida with kyphosis	1 (1.0%)	0 (0.0%)	1 (0.7%)

Gynecological examination	Not done	83 (83.8%)	33 (78.6%)	116 (82.3%)
	Yes, normal	16 (16.2%)	9 (21.4%)	25 (17.7%)

Iron profile (ferritin) levels	Not done	78 (78.8%)	36 (85.7%)	114 (80.9%)
	Yes, normal	18 (18.2%)	4 (9.5%)	22 (15.6%)
	Yes, reduced	3 (3.0%)	2 (4.8%)	5 (3.5%)

Vitamin B12 levels	Not done	79 (79.8%)	37 (88.1%)	116 (82.3%)
	Yes, normal	15 (15.2%)	2 (4.8%)	17 (12.1%)
	Yes, reduced	5 (5.1%)	3 (7.1%)	8 (5.7%)

Total cholesterol levels	Not done	88 (88.9%)	39 (92.9%)	127 (90.1%)
	Yes, normal	9 (9.1%)	3 (7.1%)	12 (8.5%)
	Yes, increased	2 (2.0%)	0 (0.0%)	2 (1.4%)

Diseases/Disorders	Hypothyroidism (treated/on treatment and under control)	5 (5.1%)	5 (11.9%)	10 (7.1%)
	Hypothyroidism (not under control)	2 (2.0%)	2 (4.8%)	4 (2.8%)
	Hyperthyroidism (treated/on treatment and under control)	2 (2.0%)	0 (0.0%)	2 (1.4%)
	Hyperthyroidism (not under control)	1 (1.0%)	1 (2.4%)	2 (1.4%)
	Not significant	89 (89.9%)	34 (81.0%)	123 (87.2%)

Gynecological history	PCOS (treated/on treatment and under control)	6 (6.1%)	1 (2.4%)	7 (5.0%)
	PCOS (not under control)	2 (2.0%)	0 (0.0%)	2 (1.4%)
	Fibroids (treated, normal)	1 (1.0%)	0 (0.0%)	1 (0.7%)
	Fibroids (not treated)	1 (1.0%)	0 (0.0%)	1 (0.7%)
	Ovarian cyst (treated, normal)	2 (2.0%)	0 (0.0%)	2 (1.4%)
	Ovarian cyst (not treated)	0 (0.0%)	0 (0.0%)	0 (0.0%)
	PCOS (on treatment), ovarian cyst (treated, normal)	1 (1.0%)	0 (0.0%)	1 (0.7%)
	Blood transfusion	0 (0.0%)	1 (2.4%)	1 (0.7%)
	Fertility treatments	1 (1.0%)	0 (0.0%)	1 (0.7%)
	None	85 (85.9%)	40 (95.2%)	125 (88.7%)

History of surgery	Tubectomy	3 (3.0%)	2 (4.8%)	5 (3.5%)
	Not significant	95 (96.0%)	40 (95.2%)	135 (95.7%)

Parity, live births, abortions	P0L0A0	55 (55.6%)	15 (35.7%)	70 (49.6%)
	P1L1A0	11 (11.1%)	6 (14.3%)	17 (12.1%)
	P1L1A1	1 (1.0%)	2 (4.8%)	3 (2.1%)
	P1L1A3	0 (0.0%)	1 (2.4%)	1 (0.7%)
	P2L2A0	28 (28.3%)	15 (35.7%)	43 (30.5%)
	P2L2A1	2 (2.0%)	0 (0.0%)	2 (1.4%)
	P3L3A0	2 (2.0%)	3 (7.1%)	5 (3.5%)

History of last/Latest pregnancy	Before 2020	36 (36.4%)	26 (61.9%)	62 (44.0%)
	In 2020	1 (1.0%)	0 (0.0%)	1 (0.7%)
	In 2021	4 (4.0%)	0 (0.0%)	4 (2.8%)
	In 2022	2 (2.0%)	1 (2.4%)	3 (2.1%)
	In 2023	1 (1.0%)	0 (0.0%)	1 (0.7%)
	None	55 (55.6%)	15 (35.7%)	70 (49.6%)

Current medications	Thyroid medications	8 (8.1%)	6 (14.3%)	14 (9.9%)
	Anticoagulants	0 (0.0%)	0 (0.0%)	0 (0.0%)
	Antiepileptics	0 (0.0%)	2 (4.8%)	2 (1.4%)
	Oral contraceptive pills (OCPs)	0 (0.0%)	1 (2.4%)	1 (0.7%)
	PCOD treatment	2 (2.0%)	0 (0.0%)	2 (1.4%)
	None/Not significant	89 (89.9%)	33 (78.6%)	122 (86.5%)

*Note:* Values expressed as mean (standard deviation) or a number (%).

**Table 2:** Onset and duration of menstrual abnormalities.

		Suspected cause	
		COVID-19 infection (*n* = 27)	Vaccination first dose (*n* = 15)
Interval between the suspected cause and onset of menstrual changes (in months)	3 months or less	19 (70.3%)	7 (46.6%)
	4–6 months	4 (14.8%)	1 (6.66%)
	7–12 months	4 (14.8%)	3 (20%)
	13–24 months	0 (0%)	4 (26.6%)

Duration of menstrual changes	3 months or less	3 (11.1%)	4 (26.6%)
	4–6 months	3 (11.1%)	6 (40%)
	7–12 months	5 (18.51%)	4 (26.6%)
	13–24 months	4 (14.8%)	1 (6.66%)
	More than 24 months	12 (44.44%)	0 (0%)

**Table 3:** Self-reported changes in menstrual parameters in participants who experienced menstrual changes after SARS-CoV-2 infection.

	**Cycles before SARS-CoV-2 infection**		**Cycles after SARS-CoV-2 infection**			
				**Due to COVID-19 infection**	**Due to vaccination first dose**	**Total**

Duration of menses	Normal (≤ 8 days)	41 (97.6%)	Reduced	2	1	3 (7.1%)
	Prolonged (> 8 days)	1 (2.4%)	Normal	22	8	30 (71.4%)
			Prolonged	1	5	6 (14.3%)
			Absent	2	1	3 (7.1%)

Frequency of cycles	Frequent (< 24 days)	2 (4.8%)	Frequent (< 24 days)	4	2	6 (14.3%)
	Normal (≥ 24 to ≤ 38 days)	37 (88.1%)	Normal (≥ 24 to ≤ 38 days)	11	5	16 (38.1%)
	Infrequent (> 38 days)	3 (7.1%)	Infrequent (> 38 days)	10	7	17 (40.5%)
			Absent (no bleeding)	2	1	3 (7.1%)

Flow volume	Light	1 (2.4%)	Light	5	2	7 (16.7%)
	Normal	39 (92.9%)	Normal	15	3	18 (42.9%)
	Heavy	2 (4.8%)	Heavy	5	9	14 (33.3%)
			Absent	2	1	3 (7.1%)

Clots	No	29 (69.0%)	No	18	8	26 (61.9%)
	Yes	13 (31.0%)	Yes, reduced	2	0	2 (4.8%)
			Yes, normal	4	4	8 (19%)
			Yes, increased	3	3	6 (14.4%)

Pain/cramps	No	14 (33.3%)	No	2	2	4 (9.5%)
	Yes	28 (66.7%)	Yes, reduced	1	2	3 (7.1%)
			Yes, normal	8	3	11 (26.2%)
			Yes, increased	16	8	24 (57.1%)

Mood swings/tension/anxiety/irritability	No	30 (71.4%)	No	20	7	27 (64.3%)
	Yes	12 (28.6%)	Yes, reduced	0	1	1 (2.4%)
			Yes, normal	3	5	8 (19%)
			Yes, increased	4	2	6 (14.3%)

Appetite changes	No	33 (78.6%)	No	16	11	27 (64.3%)
	Yes	9 (21.4%)	Yes, reduced	0	0	0 (0%)
			Yes, normal	5	2	7 (16.7%)
			Yes, increased	6	2	8 (19%)

Sleep disturbances	No	39 (92.9%)	No	20	12	32 (76.2%)
	Yes	3 (7.1%)	Yes, reduced	0	1	1 (2.4%)
			Yes, normal	1	0	1 (2.4%)
			Yes, increased	6	2	8 (19%)

*Note:* Values are expressed as number (%).

**Table 4:** Linear regression analysis on independent variables influencing the menstrual cycle parameters.

	Clots during menses		Pain/cramps during menses		Mood swings/tension/irritability		Sleep disturbances		Duration between infection/vaccine and onset of menstrual changes	
	Beta	*p* value	Beta	*p* value	Beta	*p* value	Beta	*p* value	Beta	*p* value
Age	0.036	0.748	−0.009	0.933	−0.293	0.003	0.107	0.351	−0.064	0.575
Locality	0.153	0.077	−0.028	0.743	0.002	0.974	−0.044	0.609	0.012	0.886
Occupation	−0.068	0.577	−0.156	0.198	−0.365	0.001	−0.324	0.009	0.159	0.193
BMI	−0.003	0.972	0.236	0.013	0.182	0.024	0.161	0.093	−0.172	0.072
Diet	−0.066	0.440	0.144	0.091	0.024	0.738	0.029	0.735	0.059	0.490
Duration of COVID-19 infection	0.217	0.014	0.098	0.258	0.070	0.316	0.007	0.939	−0.034	0.700
Severity of COVID-19 infection	−0.019	0.830	0.111	0.214	−0.063	0.407	0.089	0.325	−0.186	0.040

*Note:p* < 0.05 considered significant.

## Data Availability

The data supporting this study are available from the corresponding author upon reasonable request. Due to Privacy concerns, the dataset is not publicly available but can be accessed with appropriate permissions from Kempegowda Institute of Medical Sciences (KIMS) Hospital, Bengaluru.
